# Structural and Transport Properties of Hydrophilic and Hydrophobic Modified Ionomers in Proton Exchange Membrane Fuel Cells

**DOI:** 10.3390/polym16050668

**Published:** 2024-02-29

**Authors:** Qinjiang Zhang, Chao Wang, Lina Yu, Jiabin You, Guanghua Wei, Junliang Zhang

**Affiliations:** 1 SJTU Paris Elite Institute of Technology, Shanghai Jiao Tong University, Shanghai 200240, China; leon_carfield@sjtu.edu.cn; 2 Institute of Fuel Cells, Shanghai Jiao Tong University, Shanghai 200240, China; jiabinyou@163.com (J.Y.); junliang.zhang@sjtu.edu.cn (J.Z.); 3 Zhejiang TangFeng Energy Technology Co., Ltd., Huzhou 313002, China; wangchao@tangfengenergy.com; 4 Commercial Vehicle Development Institute, FAW Jiefang Automotive Co., Ltd., Changchun 130011, China; yulina@rdc.faw.com.cn; 5 MOE Key Laboratory of Power & Machinery Engineering, Shanghai Jiao Tong University, Shanghai 200240, China

**Keywords:** PEMFCs, molecular dynamics simulations, hydration level, hydrophilicity, hydrophobicity, structural and transport properties

## Abstract

The sluggish commercial application of proton exchange membrane fuel cells (PEMFCs) with low Pt loading is chiefly hindered by concentration polarization loss, particularly at high current density regions. Addressing this, our study concentrates on the ionomer membranes in the cathode catalyst layer (CCL) and explores the potential of incorporating additional hydrophilic or hydrophobic components to modify these ionomers. Therefore, an all-atom model was constructed and for the ionomer and hydrophilic and hydrophobic modifications were implemented via incorporating SiO_2_ and PTFE, respectively. The investigation was conducted via molecular dynamics (MD) simulations to predict the morphology and structure of the ionomer and analyze the kinetic properties of oxygen molecules and protons. The simulation results elaborate that the hydrophilic and hydrophobic modifications favor the phase separation and the self-diffusion coefficients of oxygen molecules and protons are enhanced. Considering the hydration level of the ionomer films, hydrophilic modification facilitates mass transfer under low-hydration-level conditions, while hydrophobic modification is more effective in optimizing mass transfer as the hydration level increases. The optimal contents of SiO_2_ and PTFE for each hydration level in this work are 9.6% and 45%, respectively. This work proposes a reliable model and presents a detailed analysis of hydrophilic and hydrophobic modifications, which provides theoretical guidance for quantitative preparations of various composite membranes.

## 1. Introduction

Proton exchange membrane fuel cells (PEMFCs) have become one of the cutting-edge topics for hydrogen energy applications due to their cleanliness, high energy conversion rate, and low noise pollution [1]. Nevertheless, the rapid development of PEMFCs and the investment in commercial use are greatly constrained due to drawbacks such as high manufacturing and operation costs and low durability [2]. The current mainstream approach to cost reduction is decreases in Pt loading. Nevertheless, insufficient Pt leads to a severe concentration polarization at high current densities, where the high local transport resistance in the cathode catalyst layer occupies more than 50% of the total loss [3,4,5]. In the cathode catalyst layer of PEMFCs, the three-phase interface formed by the compact contact of electrolytes, catalyst particles, and gas is the core place where the electrochemical reaction takes place [6]. Therefore, a detailed understanding of the changes in the internal morphology and structure within the catalyst layer, as well as the transport mechanisms of water molecules, protons, and gas molecules, is essential to optimize cell performance. 

Water distribution in PEMFCs has a crucial impact on improving the efficiency of the cell. On one hand, protons are transported in their hydrated form inside the membrane electrode, implying that the conduction depends on the moisturizing ability of the proton exchange membrane (PEM). On the other hand, excessive water can lead to flooding, obstructing the diffusion pathways of reactant gases, particularly at high power densities and flow rates where water production is elevated, yet insufficient drainage exacerbates the problem. The accumulation of liquid water can impede gas diffusion and precipitate a decline in cell performance due to exacerbated concentration polarization at the reaction sites and a decrease in the oxygen reduction reaction (ORR) rate [7]. Modification of the hydrophilicity and hydrophobicity of the cathode catalyst layer is one of the important ways to ameliorate water distribution in PEMFCs. The introduction of hydrophilic substances has been proven as an accessible but effective way. Previous experiments have demonstrated that the incorporation of SiO_2_ into the membrane increases its water uptake, indicating an enhanced hydrophilicity of the membrane [8]. Moreover, SiO_2_ tends to cover the surface of Pt particles and prevents Pt particles from displaying different decaying behaviors, thus contributing to the stability and maintenance of the battery performance [9,10,11]. Additionally, SiO_2_ enables PEMs to function properly at high temperatures due to its hygroscopic capability [12]. Other substances, like Al_2_O_3_ [13] and TiO_2_ [14], can also endow the CCL with self-humidifying abilities via mixing them with the PEM during fabrication. However, SiO_2_ has a lower procurement cost compared to TiO_2_ and a better chemical stability compared to Al_2_O_3_. Herein, SiO_2_ is more accessible in hydrophilic modifications of PEMFCs.

Researchers have found that the doping hydrophobic substance weakens the interactions between Pt and Nafion and modifies the distribution of ionomers on the surface of the Pt particles. Only weak van der Waals interactions exist between the modified Pt surface and the ionomers instead of polar interactions [15]. Polytetrafluoroethylene (PTFE) is one of the commonly used substances for hydrophobic modification of the CCL. It is reported that PTFE can create a rougher material surface, which can drastically increase the contact angle of the water droplets, resulting in a further increase in the hydrophobicity of the coating. Consequently, the number of transport channels increases and the oxygen can reach the triple phase boundary (TPB) sites more effectively [16,17]. Sun et al. [18] constructed another Pt/C hydrophobic interface by adding 1-hexadecyl methanethiol (C16SH), which successfully reduced the density of sulfonate groups surrounding the Pt active site, and obtained a specific Pt/ionophore interface after removing the C16SH by in situ electrochemical oxidation. Experimentally, according to the limiting current density method, the mass transfer resistance of oxygen at the Pt/ionophore interface was reduced and the performance of PEMFCs was enhanced.

At the current stage, the connection between the morphological changes inside the catalyst layer and the mass transfer phenomena is difficult to explain intuitively through macroscopic experiments. Thus, its simulation at the microscopic level is particularly important in the study of its mechanism. MD simulations have been widely utilized in the PEMFC field, including investigations of the effect of I/C ratios on cell performance [19], the effect of hydration level on oxygen transport [20], the proton conduction mechanism [21], etc.

Prior studies have discovered that an excessive amount of hydrophilic and hydrophobic substances may bring some negative impacts. For instance, SiO_2_ may lead to an increased flooding phenomenon, resulting in the loss of battery performance [10]. Likewise, the over-doping of PTFE will hinder the proton conduction, which is not conducive to the enhancement of the performance of PEMFCs either. Herein, a quantitative analysis of the effects of hydrophilic and hydrophobic modifications is necessary. In this work, we constructed all-atom models for different types of CCLs. Then, MD simulations were carried out which enabled us to specifically describe the structural and transport properties and finally render a visual result.

## 2. Materials and Methods

### 2.1. Modeling of PEMFC Cathode Catalyst Layer

**Figure 1:**
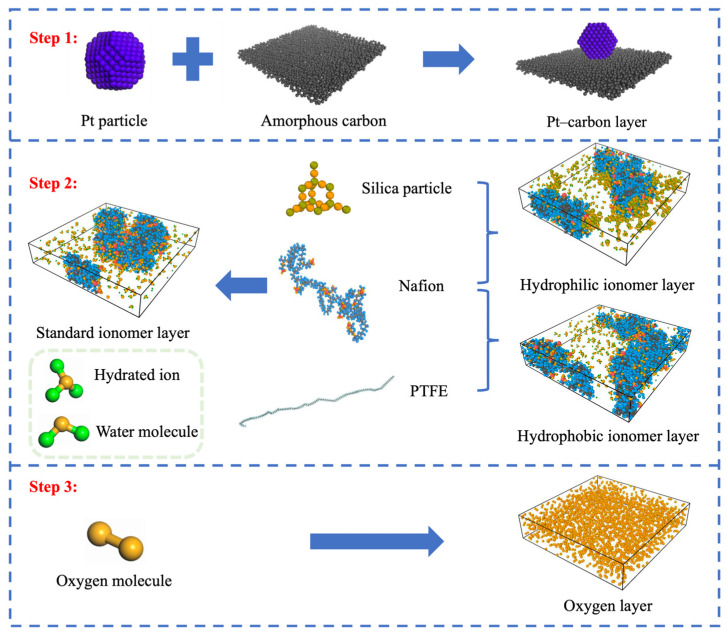
Flow chart of the model construction. The gray, purple, red, blue, green, orange, and olive spheres represent carbon, platinum, sulfur, fluorine, hydrogen, oxygen, and silicon atoms, respectively.

The all-atom molecular dynamics model was constructed with a Pt/C layer, an ionomer layer, an oxygen layer, and a vacuum layer in order from the bottom up. The specific construction process is illustrated in Figure 1. 

The amorphous carbon was used in the carbon layer to simulate the Vulcan XC-72 carbon black with a size of 70 Å × 70 Å × 6 Å. The Pt particles were cut into truncated octahedrons [22,23] with a diameter of 2.28 nm and were fixed to the carbon layer to ensure the simulation results were unaffected by Pt particle movement. The baseline ionomer layer contained 6 Nafion chains with a polymerization degree of 10, 60 hydrated ions (H_3_O^+^), and water molecules. Three different hydration levels (λ = 3, 5, 8) were set, which referred to the number of water molecules and hydrated ions per sulfonate group (-SO_3_^2−^). The hydrophilic and hydrophobic modifications were realized via adding a designed number of SiO_2_ or PTFE, respectively. The SiO_2_ content in the hydrophilic ionomer film was determined by the mass ratio of SiO_2_ to Nafion, and three gradients of 4.8%, 9.6%, and 14.5% were set, corresponding to the number of SiO_2_ particles of 5, 10, and 15, respectively. The PTFE chain used in hydrophobic ionomer membranes consisted of 100 -C_2_F_4_- monomers and the content was determined likewise by the same standard: four gradients of 15%, 45%, 75%, and 120% were set, corresponding to 1, 3, 5, and 8 PTFE chains, respectively. The oxygen layer was built according to the method mentioned in MD simulations conducted by previous studies [24]. The initial pressure in the oxygen diffusion region was set to 10 MPa to guarantee that the simulation would obtain a sufficient sample of permeable oxygen molecules in a short period. In an attempt to cancel the effect of the periodic boundary conditions on the oxygen mass transfer in the *z*-axis direction, a vacuum layer region of 100 Å thickness was added above the oxygen layer. The baseline model with different hydration levels was noted as Std (λ = 3/5/8), while other hydrophilic and hydrophobic models were labeled as SiO_2_-4.8%/9.6%/14.5% (λ = 3/5/8) and PTFE-15%/45%/75%/120% (λ = 3/5/8), respectively.

### 2.2. MD Simulation Process

The COMPASS force field [25], which is an ab initio force field capable of accurately predicting the structure and thermophysical properties of small inorganic molecules [26], was applied in the simulation. The total potential energy in the COMPASS force field was determined by Equation (1):

(1) $$E_{total}=E_{VdW}+E_{Q}+E_{bond}+E_{angle}+E_{torsion}+E_{cross-coupling}$$

where $E_{total}$, $E_{VdW}$, $E_{Q}$, $E_{bond}$, $E_{angle}$, $E_{torsion}$, and $E_{cross-coupling}$ represent the total potential energy, van der Waals interactions, electrostatic interactions, bonding potentials, angular potentials, dihedral angular twisting potentials, and cross-coupling phases, respectively. 

To acquire an equilibrium model, an MD simulation process was conducted using the Forcite modules of Materials Studio. Firstly, the ionomer layer was placed above the Pt/C layer, and geometry optimization was carried out using the steepest descent method [27]. Secondly, the model was subjected to NPT kinetic simulations at 353.15 K and 0.15 MPa for 20 ps. The simulated system was then controlled under the NVT system and four annealing operations were performed with the upper and lower temperature limits set at 1053.15 K and 353.15 K. Thirdly, the kinetic simulations were performed for 20 ps under the NVT system. The annealing and NVT kinetic simulations were repeated three times to confirm that the model reached the equilibrium state. The oxygen layer and the vacuum layer were then added to the above simulated system and the aforementioned simulation steps were duplicated. Finally, a 100 ps constant temperature kinetic simulation was performed under the NVT system to collect data for subsequent analysis of the internal structure and kinetic properties of the model.

### 2.3. Analysis Methods

To understand the microstructure in ionomer films, the radial distribution function (RDF) was used to embody precise equilibrium information. Four pairs of atoms are emphasized in this work:

- Pt with carbon atoms of Nafion backbone ($g_{Pt-C(Nafion)}$);
- Pt with sulfur atoms of sulfonate groups ($g_{Pt-S(SO_{3}^{-})}$);
- Pt with silicon atoms ($g_{Pt-Si}$);
- Pt with carbon atoms of PTFE ($g_{Pt-C(PTFE)}$).

The radius of gyration (*R_g_*), as an important structural characterization method, has become an important means of determining the morphology and size of polymers in microscopic research methods. Specifically, *R_g_* is defined as the average distance between the center of mass and the components of the polymer chains, as shown in Equation (2) [28]:

(2) $$R_{g}^{2}=\frac{1}{M}\sum_{1}^{N}m_{a}{\left(\vec{r_{a}}-\vec{r_{cm}}\right)}^{2}$$

where M represents the molecular mass of Nafion, $m_{a}$ and $\vec{r_{a}}$ are the molecular mass and position of atom a, and $r_{cm}$ is the position of the barycenter of Nafion.

To investigate the influence of the hydrophilic and hydrophobic modifications on the transport of oxygen molecules and protons, the self-diffusion coefficient was calculated via mean square displacement (MSD) and Einstein’s relation [29]. The MSD was obtained from the data collected during the 100 ps constant temperature kinetic simulation under the NVT system.

(3) $$MSD\left(t\right)=\sum_{1}^{N}{\left|r_{i}\left(t\right)-r_{i}\left(0\right)\right|}^{2}/N$$

(4) $$D=\underset{t\to \infty }{lim}MSD\left(t\right)/6t$$

where $r_{i}\left(t\right)$ and $r_{i}\left(0\right)$ denote the spatial position of molecule i at the moments t>0 and t=0, D represents the self-diffusion coefficient, and N represents the total number of particles of a certain type in the simulated system.

## 3. Results and Discussion

### 3.1. Analysis of the Equilibrium Structure

**Figure 2:**
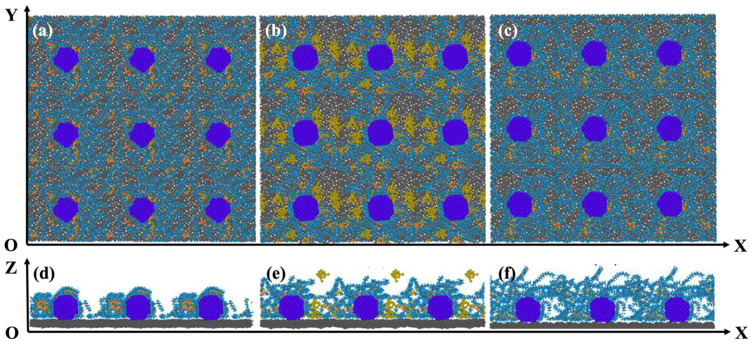
Morphology of different catalyst layers: (**a**,**d**) Std (λ = 5); (**b**,**e**) SiO_2_-9.6% (λ = 5); (**c**,**f**) PTFE-75% (λ = 5).

The model was replicated 3 × 3 in the XOY plane and cross sections in different orientations were obtained. Oxygen molecules, water molecules, and hydrated ions were removed in order to display the morphological structure inside the simulated system more intuitively. Figure 2, from left to right, sequentially represents three models with a hydration level of five: Figure 2a,d are the pure Nafion models, Figure 2b,e are the hydrophilic models, and Figure 2c,f are the hydrophobic models. In model Std (λ = 5), Nafion was more uniformly covered with Pt particles, and due to the strong interaction of the sulfonate groups of its side chains with Pt [30], Nafion became a cluster structure with the side chains inside the main chains outside. With the addition of SiO_2_ particles, the thickness of the ionomer film increased and the density decreased. Moreover, SiO_2_ tended to be distributed around the Pt particles on account of the hydrophilic interaction with Pt particles, thus occupying the original position of Nafion chains. Accordingly, there were more bare sites on the surface of the Pt particles for oxygen, hence effectively improving the ORR rate. Additionally, a larger surface of the carbon layer was exposed to oxygen compared to the baseline model. Similarly, the equilibrium configuration of PTFE-75% (λ = 5) revealed that the addition of the PTFE polymer increased the thickness of the ionomer film, with PTFE replacing some of the Nafion directly on the surface of the Pt particles. In consequence, little of the carbon substrate and Pt particles were left exposed.

**Figure 3:**
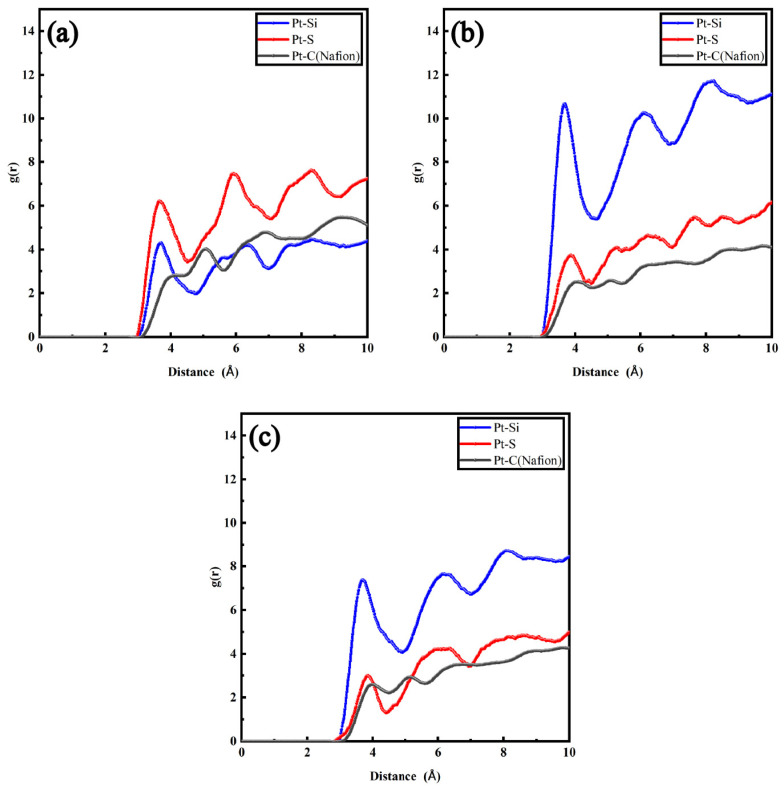
RDFs of different atom groups in hydrophilic catalyst layers: (**a**) SiO_2_-4.8% (λ = 5); (**b**) SiO_2_-9.6% (λ = 5); (**c**) SiO_2_-14.5% (λ = 5).

**Figure 4:**
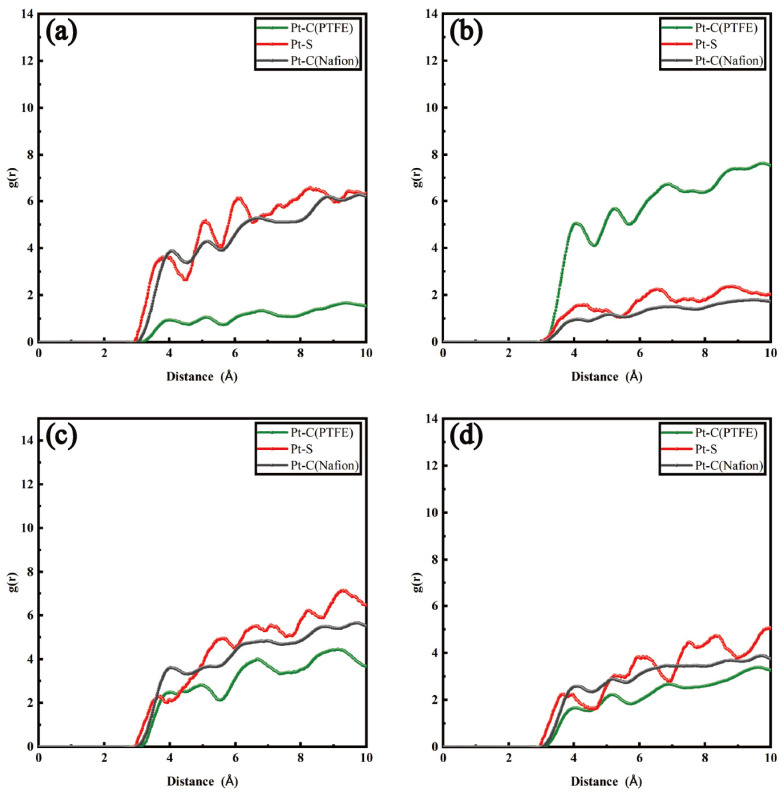
RDFs of different atom groups in hydrophobic catalyst layers: (**a**) PTFE−15% (λ = 5); (**b**) PTFE−45% (λ = 5); (**c**) PTFE−75% (λ = 5); (**d**) PTFE−120% (λ = 5).

To acquire more detailed equilibrium information, the RDFs were considered, as illustrated in Figure 3 and Figure 4. Figure 3 exhibits the RDFs of Pt particles with other atoms inside the hydrophilic catalyst layer with a hydration level λ = 5. The peak value of $g_{Pt-S(SO_{3}^{-})}$ appears nearer to Pt than that of $g_{Pt-C(Nafion)}$, which corresponds to the morphological structure illustrated in Figure 2. Among different amounts of SiO_2_ particles, the curve $g_{Pt-Si}$ of SiO_2_-9.6% (Figure 3b) showed the highest peak value, which was 12.66, indicating that a large amount of SiO_2_ particles were attracted to each other to form a large irregular cluster structure. The porosity of the ionomer became higher, shaping a sparser internal structure. The peak values of SiO_2_-14.5% (Figure 3c) and SiO_2_-4.8% (Figure 3a) decreased in order, and were 9.51 and 6.18, respectively. Actually, due to the limited ability of mutual attraction, the increase in the number of SiO_2_ particles resulted in the formation of several larger clusters, and the aggregation of hydrophilic phases was intensified. In addition, the clusters were inclined to shift away from Pt particles as elaborated in Figure 3c. The increase in the size of SiO_2_ clusters made it harder to continuously adsorb Pt particles. In the meantime, the peak of $g_{Pt-C(Nafion)}$ re-approached the Pt particles, indicating a recoating of Nafion, which was not conducive to the diffusion of oxygen in the ionomer film.

Analogously, a similar tendency was discovered after hydrophobic modifications. In Figure 4b, model PTFE−45% exhibits the highest peak value of $g_{Pt-C(PTFE)}$ with a value of 8.67, and the peak occurred closest to the Pt particles. By contrast, the peak values of the curves $g_{Pt-C(Nafion)}$ and $g_{Pt-S(SO_{3}^{-})}$ were lower. The peaks appeared far away from the surface of the carbon layer. PTFE was more concentrated around the Pt particles compared to the other hydrophobic models. The original places of Nafion were substituted, leaving more exposed active sites, which indicated that the appearance of PTFE chains forced the Nafion chains far away from the surface of the Pt particles, and the structure of the ionomer film became looser, which was favorable for the diffusion of oxygen molecules inside the ionomer film. When the content of hydrophobic molecules continued to increase from 75% to 120%, the degree of encapsulation of PTFE molecules on the surface of Pt particles declined under the hydrophobic–hydrophobic interactions between the PTFE and the carbon layer. As shown in Figure 4c,d, the peak value of $g_{Pt-C(Nafion)}$ decreased and the peaks were located far away from the Pt particles. The Nafion molecules reformed a dense ionomer film on the surface of the Pt particles, which prevented oxygen molecules from reaching the Pt particles.

**Figure 5:**
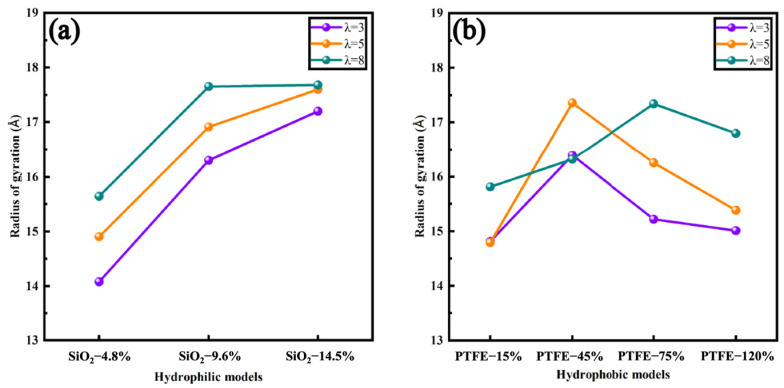
*R_g_* of carbon atoms on the backbone of Nafion: (**a**) hydrophilic models; (**b**) hydrophobic models.

Not only was the distribution of Nafion affected by hydrophilic and hydrophobic modifications, but the properties of Nafion itself were altered. In this work, the radius of gyration (*R_g_*) of the backbone of Nafion was studied, as illustrated in Figure 5. A general ascending trend of *R_g_* was found as the hydration level of the ionomer increased, which signified an elevated water content led to the expansion of Nafion. Furthermore, the water in Nafion also served as a plasticizer, enhancing the flexibility of the polymer. This increased flexibility allowed the Nafion chains to move and unfold more easily in a moist environment, further augmenting the radius of gyration. In the meantime, SiO_2_ and PTFE both contributed to an amelioration in *R_g_* because they facilitated the micro-separation of hydrophilic and hydrophobic phases. Separately, SiO_2_ particles embedded themselves into Nafion, promoting the unfolding of the backbone of Nafion, while the incorporation of PTFE into Nafion introduced additional rigidity and structural integrity to the composite material. Moreover, the hydrophobicity of PTFE altered the swelling dynamics, which led to changes in the spatial arrangement of the Nafion chains, potentially increasing their radius of gyration. Actually, researchers have discovered that the mobilities of Nafion have non-negligible impacts on transport properties [31,32]. Nafion with high backbone mobilities exhibits a less-clustered agglomerate morphology and a proper separation of microphases, which demonstrates the feasibility of hydrophilic and hydrophobic modifications. 

### 3.2. Dynamic Properties

**Figure 6:**
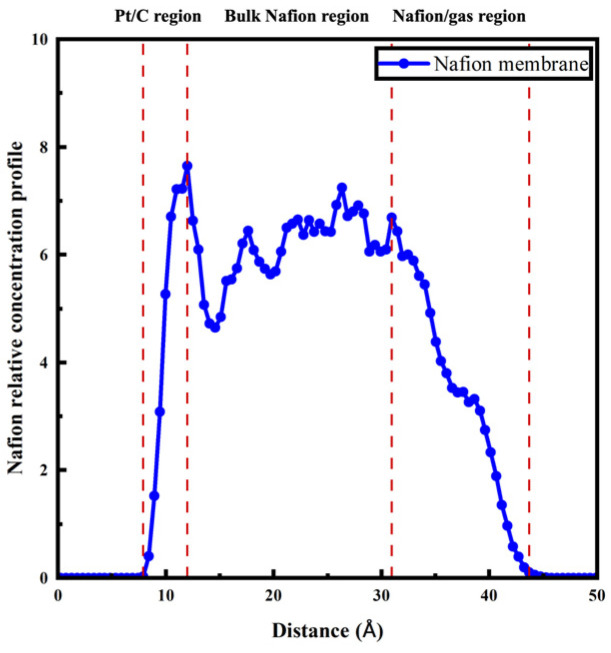
Relative concentration profile of Std (λ = 5) and division of different regions.

In order to obtain profound comprehension of the dynamic properties, the catalyst layer was divided into three regions based on the relative density distribution of the *z*-axis (Table 1): the Pt/C surface region, the bulk Nafion region, and the gas/Nafion region. For instance, the division of different regions of Std (λ = 5) is illustrated in Figure 6. 

**Figure 7:**
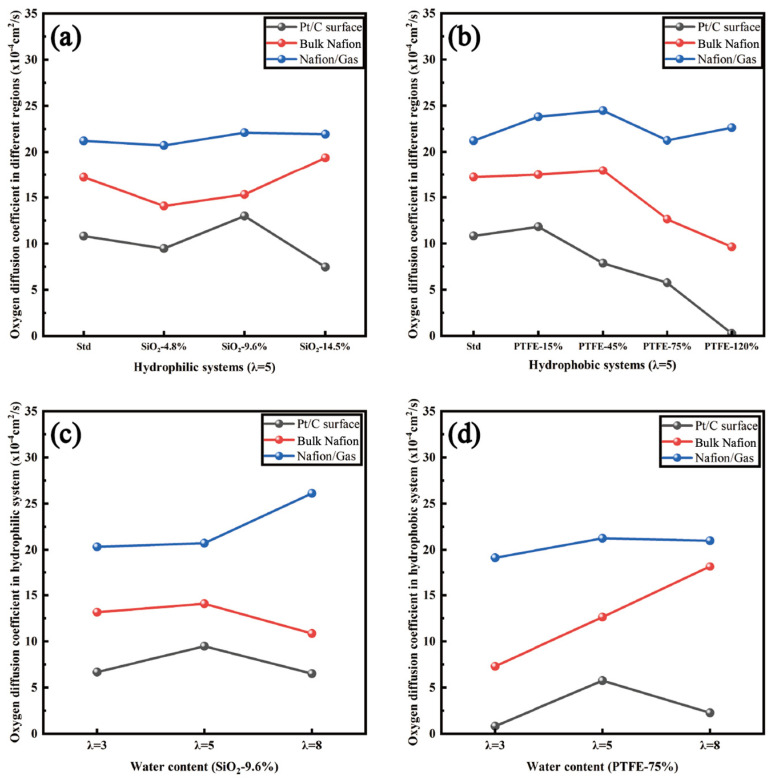
Self-diffusion coefficient of oxygen molecules influenced by various factors: (**a**) SiO_2_ content (λ = 5); (**b**) PTFE content (λ = 5); (**c**) water content in hydrophilic models; (**d**) water content in hydrophobic models.

The self-diffusion coefficients of oxygen molecules in different regions of the hydrophilic and hydrophobic models are depicted in Figure 7. As expected, the mass transfer rate of oxygen molecules decreased significantly as they approached the Pt surface. Experiments and simulations demonstrated that the dense ionomer film adjacent to the Pt surface impeded oxygen from reaching the TPB sites, thus becoming the dominant part of local oxygen transport resistance [33,34]. Additionally, the inhibition effect of the ionomer has been proven to arise from the specific adsorption of sulfonate groups and Pt particles [35,36]. Accordingly, the change in the oxygen transfer rate in bulk Nafion regions and Pt/C surface regions should be given great importance. The simulation results exhibit a similar trend in both hydrophilic and hydrophobic models. The diffusion ability of oxygen molecules tended to be enhanced and then weakened with the increase in SiO_2_ particles or PTFE content, and the optimal contents of additives were SiO_2_-9.6% and PTFE-45%, respectively. Normally, oxygen molecules diffuse mainly through two types of regions: the interface region between the hydrophilic and hydrophobic domain, and the free volume created by ionomer and Pt particles [37]. Therefore, a moderate amount of SiO_2_ particles or PTFE not only promoted the separation of hydrophilic and hydrophobic phases but contributed to a porous intramembrane structure, thus reducing the Knudsen diffusion resistance [38]. However, excessive additives hindered the diffusion process of oxygen in different ways. On the one hand, the over-doping of SiO_2_ particles resulted in an over-concentration of hydrophilic phases. Water droplets continued building up to the point of clogging the gas transport channels. On the other hand, excessive amounts of PTFE aggregated in the vicinity of the Pt and carbon layer, hence reforming a dense film and diminishing the number of active sites. 

Figure 7c,d exhibit the effect of different hydration levels on hydrophilic and hydrophobic models. A better performance of oxygen diffusion ability could be observed in hydrophilic models with low humidity, while hydrophobic models were better alternatives for oxygen transport with the increase in the hydration level. Studies have found that the increase in relative humidity will weaken the adsorption of oxygen on the gas/Nafion surface, but will enhance the diffusion of oxygen inside the bulk Nafion regions [39,40]. Therefore, possible explanations can be deduced that under low humidity conditions, SiO_2_ particles favor the diffusion process via adsorbing water molecules by their hydrophilicity compared with PTFE, thus retaining sufficient transport channels for oxygen molecules. When the hydration level is augmented, PTFE can still guarantee the adsorption rate while providing a loose intramembrane structure. Hence, hydrophobic modifications are more suitable for high-humidity membranes.

**Figure 8:**
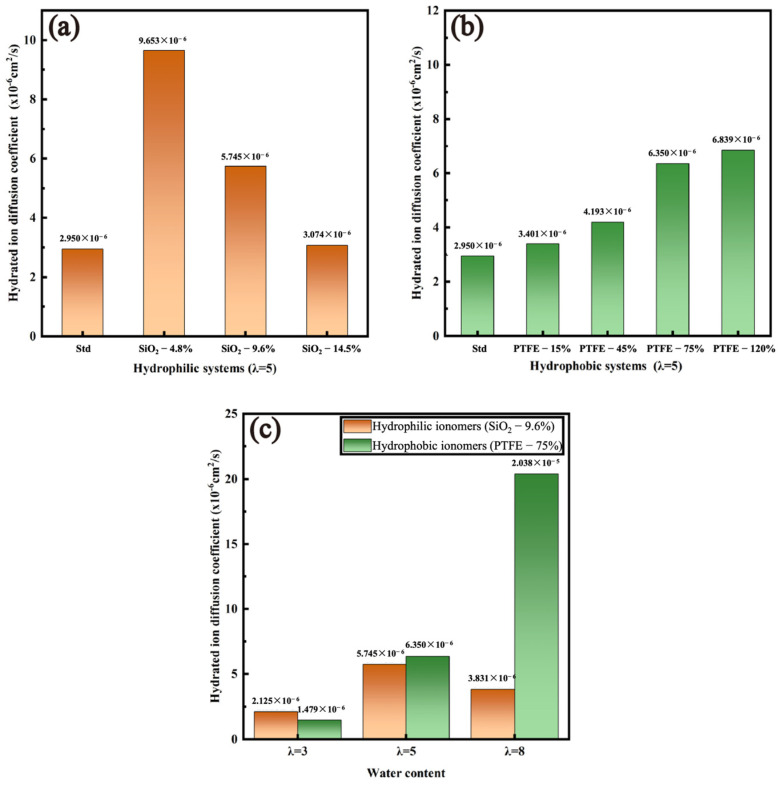
Self-diffusion coefficient of hydrated ions influenced by various factors: (**a**) SiO_2_ content (λ = 5); (**b**) PTFE content (λ = 5); (**c**) water content.

As illustrated in Figure 8, both hydrophilic and hydrophobic modifications improved the diffusion ability of hydrated ions. Specifically, the self-diffusion coefficient of hydrated ions decreases with the increasing SiO_2_ content. By contrast, the diffusion ability of hydrated ions generally increases with the increase in hydrophobicity, and this pattern is consistent with other researchers’ studies on the enhancement of the diffusion ability of hydrated ions by hydrophobic ionomer films [15]. Meanwhile, the conduction rate of hydrated ions increased continuously with increasing hydration levels, matching the pattern summarized by previous studies [41,42]. In addition, the results show that the conduction rate of hydrated ions in hydrophobic ionomer films was higher than that in hydrophilic ionomer films, and the gap widened as the hydration level kept increasing. Researchers have found that the vehicular mechanism plays a dominant role under low-hydration-level conditions, while the “Grotthus” hopping mechanism becomes more important under high hydration levels [43,44]. In concrete terms, protons are mainly conducted as H(H_2_O)_n_^+^ (i.e., H_3_O^+^, H_5_O_2_^+^, H_9_O_4_^+^) through water transport channels inside low-hydration-level membranes. Therefore, at a certain hydration level, excessive SiO_2_ would immobilize water molecules and hydrated ions in the membrane structure and lead to an incomplete hydrogen-bond network, which would reduce the rate of proton conduction. Nevertheless, in high-hydration-level membranes, hydrophobic particles attenuated the polar interactions between the water molecules and Pt particles and facilitated the formation of complete hydrogen-bond networks. In consequence, the conduction rate of hydrated ions could be ameliorated. 

## 4. Conclusions

In this research, a molecular dynamics model of PEMFC cathode catalyst layers with different hydration levels was constructed using Materials Studio, focusing on the impact of hydrophilic and hydrophobic modifications of ionomer films by adding a hydrophilic substance, SiO_2_, or a hydrophobic substance, PTFE. The addition of SiO_2_ improved the connectivity between water molecules, thus creating more transport channels for protons, while the addition of PTFE weakened the strong adsorption between water molecules and Pt particles. Moreover, the addition of SiO_2_ or PTFE enabled Nafion to unfold and enhance the mobilities, resulting in a better microphase separation. Additionally, an appropriate amount of SiO_2_ or PTFE could improve the self-diffusion coefficient of oxygen, whereas excessive SiO_2_ or PTFE would block the oxygen transport channels and thus hinder oxygen transport. In the meantime, both hydrophilic and hydrophobic modifications were able to enhance the proton conductivity in the ionomer films. The optimal contents of SiO_2_ and PTFE for each hydration level in this work are 9.6% and 45%, respectively, and these quantitative results provide a theoretical basis for further studies.

## Figures and Tables

**Table 1:** Basis for the regional division of the cathode catalyst layer.

Regions	Division Basis
Pt/C surface region	The region between the location where the relative density of the ionomer film increases from the surface of the carbon layer in the *z*-axis direction to 0.1 and the location where the first peak in the density of the ionomer film occurs.
Bulk Nafion region	The region between the location of the first peak in the density of the ionomer film and the location of the last peak.
Nafion/gas region	The area between the location where the first peak in the density of the ionomer film occurs and the location where the relative density of the ionomer film is less than 0.1.

## Data Availability

Data are contained within the article.
